# Incidence of the V600K mutation among melanoma patients with BRAF mutations, and potential therapeutic response to the specific BRAF inhibitor PLX4032

**DOI:** 10.1186/1479-5876-8-67

**Published:** 2010-07-14

**Authors:** Jill C Rubinstein, Mario Sznol, Anna C Pavlick, Stephan Ariyan, Elaine Cheng, Antonella Bacchiocchi, Harriet M Kluger, Deepak Narayan, Ruth Halaban

**Affiliations:** 1 Department of Pathology, Yale University School of Medicine, New Haven, CT 06520, USA; 2 Section of Medical Oncology, Yale University School of Medicine, New Haven, CT 06520, USA; 3 Department of Medical Oncology, New York University, New York City, New York 10016, USA; 4 Plastic and Reconstructive Surgery, Yale University School of Medicine, New Haven, CT 06520, USA; 5 Department of Dermatology, Yale University School of Medicine, New Haven, CT 06520, USA

## Abstract

Activating mutations in BRAF kinase are common in melanomas. Clinical trials with PLX4032, the mutant-BRAF inhibitor, show promising preliminary results in patients selected for the presence of V600E mutation. However, activating V600K mutation is the other most common mutation, yet patients with this variant are currently excluded from the PLX4032 trials. Here we present evidence that a patient bearing the BRAF V600K mutation responded remarkably to PLX4032, suggesting that clinical trials should include all patients with activating BRAF V600E/K mutations.

## Commentary

BRAF is a serine/threonine protein kinase, encoded on chromosome 7q34, that activates the MAP kinase/ERK-signaling pathway (see KEGG Pathways Database). Approximately 42% of melanomas harbor activating BRAF mutations (see COSMIC Database, Wellcome Sanger Trust). Most commonly, the valine at amino acid 600 is replaced by glutamate (V600E) through mutation of a single nucleotide (GTG to GAG). Another known mutation at this site involves two nucleotides (GTG to AAG), substituting lysine for valine (V600K) (see Table 1 for statistics on the frequency of these mutations reported in melanoma). The specific BRAF inhibitor PLX4032 (Plexxikon Inc., Berkeley, CA) suppresses the activated oncogenic pathway by inhibiting the ERK kinase cascade. High objective response rates were observed in the phase I clinical trial of PLX4032 in the cohort of melanoma patients selected for tumors with the V600E mutation, and ongoing phase II and phase III clinical trials are limited to those patients with BRAF V600E mutations [1].

**Table 1:** Incidence of V600 mutations in melanoma patients.

Total V600 mutants	V600E (%)	V600K (%)	V600 D or V600R (%)	Reference	Assay
34	25 (73.5)	4 (11.8)	5 (14.7)	Spittle *et al*. [7]	Pyrosequencing, validated by Sanger dideoxy sequencing

10	9 (90.0)	1 (10.0)	0 (0.0)	Hay *et al*. [8]	Melting point analysis, validated by Sanger dideoxy sequencing

44	34 (77.3)	9 (20.5)	1 (2.3)	Willmore-Payne *et al*. [9]	Amplicon melting analysis, validated by Sanger dideoxy sequencing

42	29 (69)	12 (28.6)	1 (2.3)	Halaban *et al*. [5] and Halaban, unpublished	Sanger dideoxy sequencing

50	47 (94.0)	3 (6.0)	0 (0.0)	Ugurel *et al*. [10]	Fluorescent capillary SSCP technique

178	143 (80.3)	29 (16.3)	6 (3.4)	**Total Mutations**	

The incidence of V600K mutations in melanoma may be greater than previously assumed. In our series of 138 melanomas isolated from patients with disease of varying stage, 42 harbored BRAF mutations (determined by Sanger dideoxy sequencing). Of these, 69% carried the V600E mutation (15 homozygous), while the remaining 28.6% carried the V600K variant (8 homozygous), and one carried the V600R (AGG/AGG) mutation. Of note, none of the samples are V600E/V600K heterozygotes, meaning that the V600K mutation did not arise from a second alteration at the site of an existing V600E. Altogether, the combined studies show that BRAF V600K mutations are present in 6-30% of melanoma tumors (Table 1). This broad range cannot be explained by variation in the ratio of primary versus metastatic melanomas or by the different methods used for sequencing (Table 1). Although different methods to detect the mutation were used, most of the studies validated the observations by Sanger dideoxy sequencing. Curiously, the BRAF mutations present in about 84% of nevi are reported to be of the V600E type [2,3]. Likewise, in our cohort of 14 congenital nevi, we also detected four with BRAF mutations, all heterozygous V600E. Therefore, it is possible that the V600E and V600K mutant melanomas arise from precursor lesions.

PLX4032 is a small molecule inhibitor targeting the activated form of BRAF [4]. In our recent studies on the effects of PLX4032, we demonstrated that the high enzymatic activity of both V600E and V600K BRAF mutants in melanoma cells is suppressed by treatment with PLX4032 [5]. PLX4032 is also known for its paradoxical effect on cells with wild-type BRAF, in which RAF1 is activated (reviewed in [6]). We showed that in BRAF wild-type melanoma cells, PLX4032 stimulated the downstream intracellular signaling pathway, causing cell detachment and motility in metastatic melanoma cells and enhancing cell proliferation in primary melanomas carrying NRAS Q61L mutations. These paradoxical effects highlight the importance of tailoring treatment to the specific genetic composition of the tumor [5].

**Figure 1:**
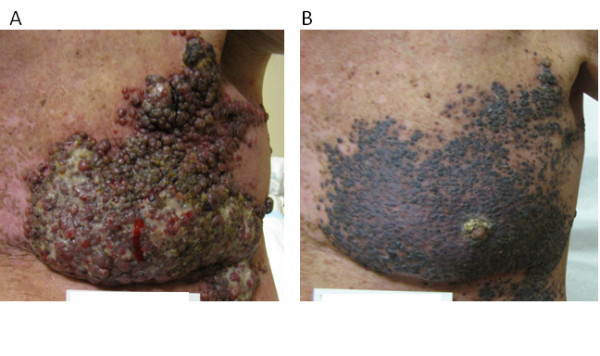
**Chest wall lesions before treatment with PLX4032 (A) and on the first day of the fifth treatment cycle (B)**.

**Figure 2:**
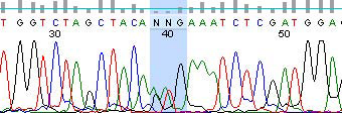
**Electropherogram from Sanger dideoxy sequencing showing the patient's melanoma tumor BRAF codon 600 mutation (AAG/GTG), encoding V600K/WT protein**.

One of our patients with locally advanced unresectable melanoma involving the skin of the left chest wall, progressing after treatment with topical imiquimod and systemic temozolomide, was referred to another institution for a phase II clinical trial of PLX4032. As part of the eligibility requirements of the trial, paraffin-embedded tissue from a biopsy of a cutaneous lesion was tested and shown to be positive for the BRAF V600E mutation. The patient was treated and achieved an excellent clinical response to PLX4032, associated with substantial reduction in the tumor burden and pain from the lesions. Figure 1 shows the cutaneous lesions before and after treatment with PLX4032. A portion of the tumor was also collected for laboratory research studies according to a protocol approved by Yale University Human Investigations Committee. Written informed consent was obtained from the patient for publication of this case report and accompanying images. A copy of the written consent is available for review by the Editor-in-Chief of this journal. Upon retesting for BRAF mutation by Sanger dideoxy sequencing, it was shown that in fact he carried the V600K allele (Figure 2, assay repeated for validation).

## Conclusions

Our data, and those of others reported in the literature, indicate that the incidence of BRAF V600K mutations in melanoma patients appears to be higher than is commonly assumed. It can be present in up to 30% of patients bearing BRAF V600 mutations, potentially representing up to 10% of all melanoma patients. Patients with BRAF V600K mutations are currently excluded from clinical trials with PLX4032, although the assay methodology used for the trial may not discriminate between the V600E and V600K mutations. Our preclinical data demonstrating similar kinase activity of the V600K and V600E mutations, together with clear evidence of clinical activity of PLX4032 in a patient with a documented V600K mutation, suggest that melanoma patients with V600K mutations should be included in current and future trials of BRAF inhibitors.

## Competing interests

The authors declare that they have no competing interests.

## Authors' contributions

JR, compiled literature search and participated in writing the manuscript; MS conceived the report and participated in writing the manuscript; ACP, treated the patient with PLX4032; SA, excised tumors and provided the material for research; EC, performed the BRAF mutation analysis; AB, collected tumor material and established melanoma cells in culture; HMK, participated in writing of the manuscript; DN, excised tumors and provided the material for research; RH, in charge of analyzing tumor specimens and participated in writing the manuscript. All authors have read and approved the final manuscript.
